# Formyl peptide receptor 2 regulates dendritic cell metabolism and Th17 cell differentiation during neuroinflammation

**DOI:** 10.3389/fimmu.2024.1354074

**Published:** 2024-08-01

**Authors:** Jong-Hyung Lim, Ales Neuwirth, Kyoung-Jin Chung, Sylvia Grossklaus, Oliver Soehnlein, George Hajishengallis, Triantafyllos Chavakis

**Affiliations:** ^1^ Laboratory of Innate Immunity and Inflammation, Department of Basic and Translational Sciences, Penn Dental Medicine, University of Pennsylvania, Philadelphia, PA, United States; ^2^ Institute for Clinical Chemistry and Laboratory Medicine, University Hospital and Faculty of Medicine, Technische Universität Dresden, Dresden, Germany; ^3^ Laboratory of Adaptive Immunity, Institute of Molecular Genetics of the Czech Academy of Sciences, Prague, Czechia; ^4^ Institute for Experimental Pathology, Center for Molecular Biology of Inflammation, University of Münster, Münster, Germany

**Keywords:** formyl peptide receptor 2, dendritic cells, Th17 cells, nitric oxide, metabolism experimental autoimmune encephalomyelitis

## Abstract

Formyl peptide receptor 2 (FPR2) is a receptor for formylated peptides and specific pro-resolving mediators, and is involved in various inflammatory processes. Here, we aimed to elucidate the role of FPR2 in dendritic cell (DC) function and autoimmunity-related central nervous system (CNS) inflammation by using the experimental autoimmune encephalomyelitis (EAE) model. EAE induction was accompanied by increased *Fpr2* mRNA expression in the spinal cord. FPR2-deficient (*Fpr2* ^KO^) mice displayed delayed onset of EAE compared to wild-type (WT) mice, associated with reduced frequencies of Th17 cells in the inflamed spinal cord at the early stage of the disease. However, FPR2 deficiency did not affect EAE severity after the disease reached its peak. FPR2 deficiency in mature DCs resulted in decreased expression of Th17 polarizing cytokines IL6, IL23p19, IL1β, and thereby diminished the DC-mediated activation of Th17 cell differentiation. LPS-activated FPR2-deficient DCs showed upregulated *Nos2* expression and nitric oxide (NO) production, as well as reduced oxygen consumption rate and impaired mitochondrial function, including decreased mitochondrial superoxide levels, lower mitochondrial membrane potential and diminished expression of genes related to the tricarboxylic acid cycle and genes related to the electron transport chain, as compared to WT DCs. Treatment with a NO inhibitor reversed the reduced Th17 cell differentiation in the presence of FPR2-deficient DCs. Together, by regulating DC metabolism, FPR2 enhances the production of DC-derived Th17-polarizing cytokines and hence Th17 cell differentiation in the context of neuroinflammation.

## 1 Introduction

Experimental autoimmune encephalomyelitis (EAE) is a well-established animal model for studying multiple sclerosis (MS), a chronic autoimmune disorder of the central nervous system (CNS) (1, 2). In EAE, infiltration of autoreactive T cells into the CNS drives inflammation, demyelination of neurons and neuroaxonal damage. Key effector cells implicated in CNS pathology of EAE and MS include IFNγ-producing T cells (Th1) and IL17-producing T cells (Th17) (3). EAE was initially thought to be driven by Th1-mediated mechanisms; however, Th17 cells, i.e. CD4^+^ T cells expressing IL17, are now considered the primary pathogenic subset of T cells in EAE (4–7).

The initiation of EAE involves the interaction of antigen-specific naïve CD4^+^ T cells with antigen-presenting dendritic cells (DCs) during the priming phase. During the primary immune response, immune cells such as DCs, B cells, and macrophages play a role in EAE development by providing costimulatory signals and secreting inflammatory cytokines (8–10). The functional differentiation of CD4^+^ T cells into Th1 and Th17 cell lineages is influenced by specific cytokines produced by activated antigen-presenting cells (11). In the presence of IFNγ and IL12, naïve CD4^+^ T cells differentiate into Th1 cells (12, 13), whereas TGFβ, IL6, IL23, and IL1β promote Th17 development (5, 14–16). Th1 and Th17 cell subsets proliferate and migrate from lymph nodes to the CNS, driving CNS inflammation, which involves recruitment of further inflammatory cells (17). Therefore, the interaction between DCs and T cells in lymphoid organs plays a crucial role in determining the character and magnitude of the ensuing T cell response.

Upon encountering signals mostly mediated by pattern-recognition receptors, immature DCs upregulate the expression of major histocompatibility complex (MHC) II and co-stimulatory molecules (CD80, CD86, and CD40), transitioning to a mature state associated with changes in cellular metabolism and the ability to secrete inflammatory cytokines (18). DCs undergo two rounds of metabolic reprogramming upon activation. Within minutes after Toll-like receptor (TLR) stimulation, the glycolytic rate of BMDCs increases rapidly and remains elevated for several hours, independent of inducible nitric oxide synthase (iNOS) signaling (19, 20). In the later stage of DC activation, the sustained glycolytic reprogramming of DCs is dependent on nitric oxide (NO), which blocks mitochondrial oxidative phosphorylation (OXPHOS) and promotes glycolysis as the energy source (20, 21). This finding indicates that NO production in mature DCs is integral to their metabolic reprogramming. Recent studies have reported an inhibitory role of NO on DC-mediated inflammation. Inhibition of iNOS enhances DC costimulatory molecule expression and inflammatory cytokine production (22, 23). Consistently, administration of a chemical NO-donor significantly reduces costimulatory molecule expression and IL12, IL6, and TNFα cytokine production in human DCs, thereby modulating CD4^+^ T cell polarization (24, 25). Moreover, NO significantly suppresses Th17 cell proliferation and differentiation; mice lacking iNOS (NOS2) develop more severe EAE with increased Th17 cell accumulation in the spinal cord, as compared to wild-type (WT) controls (26, 27).

Formyl Peptide Receptor 2 (FPR2) is a transmembrane G protein-coupled receptor primarily expressed in phagocytic leukocytes, including DCs, macrophages, and neutrophils (28, 29). FPR2 displays a broad ligand promiscuity and recognizes formylated peptides and lipid mediators derived from invading pathogens, damaged tissues, or dead cells. FPR2 has been reported to exert both pro-inflammatory and pro-resolution effects, depending on the nature of the ligand (30, 31). The role of FPRs in different inflammatory reactions has been studied (32). However, the role of FPR2 in modulating T cell responses remains less clear. It has been shown that FPR2 ligation inhibits the expansion of effector pathogenic CD4^+^ T cells in two arthritis models, whereby FPR2 signaling enhances IL17 production in CD4^+^ T cells, while leaving IFNγ unchanged (33). Additionally, *Fpr2* ^KO^ mice displayed attenuated disease severity in an allergic airway inflammation model, associated with reduced recruitment of CD11c^+^ DCs into airway mucosa and secondary lymphoid organs (34). Other studies have suggested a potential role of FPRs in modulating brain inflammation (32, 35).

Here, we sought to investigate the involvement of FPR2 in the immune response and EAE development by utilizing FPR2-deficient mice and focusing on the role of DCs. Our findings indicate that FPR2 deficiency in DCs leads to elevated NO production and decreased production of Th17 polarizing cytokines by DCs, thereby resulting in reduced Th17 differentiation and delayed EAE disease onset.

## 2 Materials and methods

### 2.1 Mice and induction of EAE

*Fpr2* ^KO^ mice in C57BL/6 background were previously described (34). All mice were maintained under specific pathogen-free conditions. Animal experiments were approved by the Landesdirektion Sachsen, Germany and by the Institutional Animal Care and Use Committee of the University of Pennsylvania. EAE was induced in eight to twelve-weeks-old female mice, by using the myelin oligodendrocyte glycoprotein 35–55 (MOG_35-55_) peptide (American Peptide Company, Sunyvale, CA, USA and Genemed Synthesis Inc., San Antonio, TX, USA) model (36, 37). In the course of EAE, mice were scored daily according to the following scale: 0, no clinical sign; 1, limp tail or hind limb weakness; 2, limp tail and hind limb weakness; 3, limp tail and unilateral hind limb paralysis; 4, limp tail and bilateral hind limb paralysis; 5, four leg paralysis; and 6, moribund or dead. For disease scoring, mice that maintained a clinical score of 1 or higher for at least two consecutive days were included (38).

### 2.2 Hematoxylin & eosin staining

Spinal cords were collected from euthanized mice after systemic perfusion and fixed in 4% paraformaldehyde overnight, followed by immersion in 10% and 30% sucrose in PBS stepwise. The tissue was embedded in Optimal Cutting Temperature (OCT) media. Ten μm sections of the spinal cords were stained with hematoxylin and eosin (H & E). Images of the sections were collected using a Nikon Eclipse Ni-E microscope.

### 2.3 Isolation of leukocytes and flow cytometry analysis

Spinal cord was collected from euthanized mice after systemic perfusion and a single-cell suspension was prepared by passing through a 100 µm cell strainer (39–41). In order to obtain a leukocyte-enriched spinal cord fraction, myelin was removed using Myelin Removal Beads II (Miltenyi Biotec, Bergisch Gladbach, Germany). Spinal cord leukocytes were pre-incubated with anti-mouse CD16/32 (Fc block; BD Biosciences, Heidelberg, Germany) and stained with the following antibodies using separate panels for myeloid and lymphoid cells: anti-CD45-PerCP (Biolegend, clone 30-F11), anti-CD11b-PeCy7 (Biolegend, clone M1/70), anti-Ly6G-PE (Biolegend, clone 1A8), anti-F4/80-AF488 (Biolegend, clone BM8), anti-CD11c-APC (Biolegend, clone N418), anti-CD4-AF488 (Biolegend, clone GK1.5) and anti-TCRβ-Pe-Cy7 (Biolegend, clone H57–597). For the analysis of leukocytes at day 7 post-immunization, single cell suspensions were prepared from inguinal draining lymph nodes (dLN) using a 70 µm strainer. Following antibodies were used: anti-CD45-Pacific Blue (Biolegend, clone 30-F11), anti-CD4-APC-Cy7 (Biolegend, clone GK1.5), anti-CD8a-PerCP (Biolegend, clone 53–6.7), anti-CD11c-APC (Biolegend, clone N418), anti-F4/80-FITC (Biolegend, clone BM8), anti-CD19-PE (Biolegend, clone 6D5).

### 2.4 Ex vivo recall assay

Leukocytes isolated from inflamed spinal cords or dLNs from EAE mice at different days post-immunization, as described in the figure legends, were re-stimulated with MOG_35-55_ (25 µg/ml) overnight at 37°C, and treated with Brefeldin A for the last 4 h. To detect IL17A or IFNγ secretion, culture supernatants were collected prior to Brefeldin A treatment, and stored at -80°C until analysis. The cells were stained with anti-CD4-eFluor 450 (Thermofisher, clone GK1.5) or anti-CD4-PE (Biolegend, clone GK1.5) prior to fixation and permeabilization step using Foxp3 intracellular staining kit (eBioscience, Frankfurt, Germany). APC-conjugated antibody to IL17A (Biolegend, Clone TC11–18H10.1) or FITC-conjugated antibody to IL17A (Biolegend, Clone TC11–18H10.1) and AF488-conjugated antibody to IFNγ (Biolegend, XMG1.2) or APC-conjugated antibody to IFNγ (Biolegend, XMG1.2) were used. Cell suspension from dLNs was stained with Zombie aqua dye (Zombie Violet™ Fixable Viability kit, Biolegend) to distinguish between live and dead cells.

### 2.5 Bone marrow-derived dendritic cells (BMDCs)

Bone marrow cells were isolated from femurs of mice, and cells were then incubated in complete RPMI1640 medium supplemented with 20 ng/ml GM-CSF (PeproTech, Rock Hill, NJ, USA) and 20 ng/ml IL4 (PeproTech, Rock Hill, NJ, USA). Half of the culture medium was refreshed every 3 days and on day 7–9, the non-adherent cells were harvested and CD11c^+^ cells were isolated by CD11c microbeads (Miltenyi Biotech) and seeded for subsequent experiments. To stimulate BMDCs, 100 ng/ml LPS (Escherichia coli serotype 0111:B4, Sigma-Aldrich) was added and incubated for different time points (3 h, 6 h or overnight). For analysis of BMDC activation, cells were treated with 100 ng/ml LPS for 3 hours, and flow cytometry was conducted using the following antibodies: anti-CD80-eFluor450 (eBioscience, Clone 16–10A1), anti-CD86-Pe-Cy7 (BD Bioscience, Clone GL1), anti-I-A^b^-FITC (Biolegend, Clone AF6–120.1), anti-CD11c-APC (Biolegend, Clone N418). To detect mitochondrial superoxide levels, flow cytometry was performed using MitoSOX Red superoxide indicator (Invitrogen) at a final concentration of 5 μM, according to the manufacturer’s instructions. The mitochondrial membrane potential was quantified by flow cytometry using tetramethylrhodamine ethyl ester (TMRE, Invitrogen) at a final concentration of 100 nM, according to the manufacturer’s instructions. For studies related to involved signaling pathways, BMDCs were pre-treated for 30 mins with MEK/ERK pathway inhibitor (U0126, 10 μM, Sigma-Aldrich) or PI3K/Akt inhibitor (LY294002, 10 μM, EMD Millipore Corporation), or vehicle control (DMSO), followed by LPS treatment for 3 hours and downstream analysis.

### 2.6 *In vitro* Th17 cell differentiation

Naïve CD4^+^ T cells (CD4^+^CD62L^high^) were isolated from WT mice using a cell sorter (FACSAria, BD Biosciences). PE-conjugated antibody to CD4 (eBioscience, GK1.5) or PerCP-conjugated antibody to CD62L (Biolegend, MEL-14) were used. For DC-mediated Th17 cell differentiation, BMDCs were co-cultured with the naïve CD4^+^ T cells in the presence of purified anti-CD3 antibody (0.3 µg/ml) (145–2C11, Biolegend), human TGFβ1 (3 ng/ml) (Biolegend), and LPS (100 ng/ml) (Escherichia coli serotype 0111:B4, Sigma-Aldrich) for 4 days. Cells were re-stimulated with PMA (50 ng/ml) (Sigma-Aldrich) and Ionomycin (1 µg/ml) (Sigma-Aldrich) for 4 hours in the presence of Brefeldin A solution (eBioscience). Cells were harvested and blocked with 1 µg/ml of Fcγ block (BD, Heidelberg, Germany) for 10 mins at 4°C in FACS buffer (PBS containing 0.1% BSA and 0.1% NaN3). The cells were stained with PE-conjugated CD4 for 30 mins at 4°C, washed twice with permeabilization buffer, fixed, permeabilized in a fixation/permeabilization buffer (eBioscience, Frankfurt, Germany) for 40 mins at 4°C, stained with FITC-conjugated IL17A (Biolegend, Clone TC11–18H10.1) for 30 min at 4°C and then analyzed by flow cytometry. For NO inhibition assay, 500 µM S−ethylisothiourea (SEITU) (Cayman Chemical) was added to the co-culture plate.

### 2.7 Quantitative real-time PCR (qRT PCR)

Total RNA was extracted using a RNeasy plus kit (QIAGEN) or Trizol (Invitrogen, Carlsbad, CA), and cDNA was generated with an oligo(dT) primer and the iScript cDNA kit and then the resulting cDNAs were subjected to real-time PCR amplification with SsoFast EvaGreen Supermix kit (Bio-Rad, Munich, Germany) by using Bio-Rad CFX 384 Real-Time System (Bio-Rad, Munich, Germany) or the Applied Biosystems 7500 Fast Real-Time PCR System according to the manufacturer’s protocol (Life Technologies). Data were analyzed using the comparative (ΔΔCt) method. *18S* or *Gapdh* was used as an internal control. Primer sequences used are listed as follows; *Il23p19* (Forward: 5’-AGCGGGACATATGAATCTACTAAGAGA-3’, Reverse: 5’-GTCCTAGTAGGGAGGTGTGAAGTTG-3’), *Nos2* (Forward- 5’-CCTGCTTTGTGCGAAGTGTC-3’, Reverse: 5’-CCTCCTTTGAGCCCTTTGTG), *Il1b* (Forward: 5’- ATCCCAAGCAATACCCAAAG-3’, Reverse: 5’-GTGCTGATGTACCAGTTGGG-3’), *Il17a* (Forward: 5’-CGCAAAAGTGAGCTCCAGA-3’, Reverse: 5’-TGAGCTTCCCAGATCACAGA-3’), *18S* (Forward: GTTCCGACCATAAACGATGCC-3’, Reverse: 5’-TGGTGGTGCCCTTCCGTCAAT-3’), *Fpr2* (Forward: 5’- CTGAATGGATCAGAAGTGGTGG-3’, Reverse: 5’-CCCAAATCACTAGTCCATTGCC-3’), *Il6* (Forward: 5’- CCTTCCTACCCCAATTTCCAAT-3’, Reverse: 5’-AACGCACTAGGTTTGCCGAGTA-3’). TaqMan probes and gene-specific primers for detection and quantification of mouse genes investigated in certain experiments were purchased from Thermo-Fisher Scientific and included: *Fpr2* (Mm00484464_s1), *Nos2* (Mm00440502_m1), *Aco2* (Mm00475673_g1), *Idh1* (Mm00516030_m1), *Idh3a* (Mm00499674_m1), *Suclg1* (Mm00451244_m1), *Dlat* (Mm00455160_m1), *Ogdh* (Mm00803119_m1), *Fh1* (Mm01321349_m1), *Ndufa9* (Mm00481216_m1), *Sdhb* (Mm00458272_m1), *Uqcrc2* (Mm00445961_m1), *Atp5b* (Mm01160389_g1), *Cox2* (Mm03294838_g1), *Gapdh* (Mm99999915_g1).

### 2.8 Seahorse analysis

BMDCs (7x10^4^) were plated in 50 μl XF Seahorse medium (Agilent, Santa Clara, CA, USA) (pH 7.4) supplemented with 2 mM glutamine, 10 mM glucose and 2 mM sodium pyruvate. Plates were centrifuged at 200 g without break and cells were then incubated for 30 min at 37°C without CO_2_. Afterwards, 130 μl of the same XF medium was added to each well. Real-time measurements of oxygen consumption rate (OCR) were obtained as indicated by the manufacturer’s instructions. OCR was measured at basal conditions and after sequential stimulation of the cells with 1 μM Oligomycin, 1 μM FCCP and 0.5 μM Rotenone/Antimycin (all included in the Mitostress kit, Agilent, Waldbronn, Germany) in a Seahorse XFe96 Analyzer (Agilent) using the Wave Software (Agilent). The measurements were normalized by the DNA content of the cells, assessed by using the CyQUANT cell proliferation kit (Invitrogen).

### 2.9 Measurement of cytokines by ELISA

Analysis of IL6, IL1β, IL23, IFNγ, IL17A in culture supernatants was performed with the DuoSet ELISA kit (R&D Systems) or Mouse Uncoated ELISA kit (Invitrogen), according to manufacturer’s description.

### 2.10 Nitric oxide assay

BMDCs were incubated overnight post treatment with LPS at 37°C and 5% CO_2_ and total nitrite levels in the cell culture supernatant were determined using Nitric oxide and Nitrate/Nitrite Assay kit (R&D system).

### 2.11 Statistical analysis

Statistical analysis was performed with Prism software (Graphpad, San Diego, CA). Unpaired Student’s *t* test was used for comparisons between two groups. Two-way ANOVA with Sidak’s multiple comparison test was used for clinical score analysis. One-way ANOVA with Tukey’s multiple comparison test or Dunnett’s multiple comparison test was used for comparisons among multiple groups. P < 0.05 was considered significant.

## 3 Results

### 3.1 Delayed EAE disease onset in *Fpr2^KO^* mice

**Figure 1:**
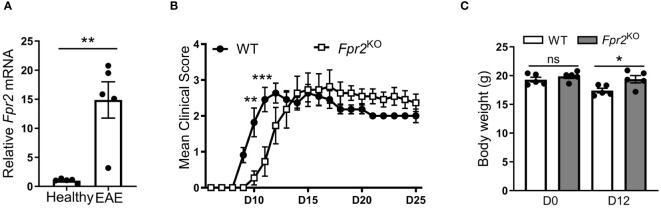
Delayed onset of experimental autoimmune encephalomyelitis (EAE) in *Fpr2* ^KO^ mice. EAE was induced in mice by MOG_35-55_ peptide immunization. **(A)** Relative *Fpr2* mRNA expression in the spinal cord of healthy WT mice or WT mice subjected to EAE (at the peak of disease; day 19 post-immunization), as assessed by qRT-PCR. The mRNA expression was normalized to *18S* and the gene expression of WT mice was set as 1 (n=5 mice per group). **(B)** Clinical EAE score in WT and *Fpr2* ^KO^ mice (n=11 mice per group); D: day. **(C)** Body weight of WT and *Fpr2* ^KO^ mice at day 0 (healthy) or upon EAE induction (day 12 post-immunization) (n=5 mice per group); D: day. Data are mean ± SEM. * p< 0.05, ** p<0.01, *** p<0.001; ns, not significant.

To investigate the role of FPR2 in CNS inflammation, we initially monitored the expression of *Fpr2* mRNA in the spinal cord during EAE. We observed a significant upregulation of *Fpr2* expression in the spinal cord both at the early stage and the peak of EAE, as compared to healthy mice (**Supplementary Figure 1**, **Figure 1A**). We next assessed the role of FPR2 in EAE development. Compared to WT littermate controls, *Fpr2* ^KO^ mice exhibited delayed EAE disease onset, as illustrated by the significantly reduced severity at the early stage of the disease (**Figure 1B**); however, no difference was observed in EAE severity, after the disease reached its peak. Consistent with the reduced disease severity at the early phase of EAE, *Fpr2* ^KO^ mice exhibited higher body weight, compared to the WT controls (**Figure 1C**). Together, these findings suggest that FPR2 plays a role in the initiation phase of EAE.

### 3.2 FPR2-deficiency is associated with a decreased Th17 response during EAE development

**Figure 2:**
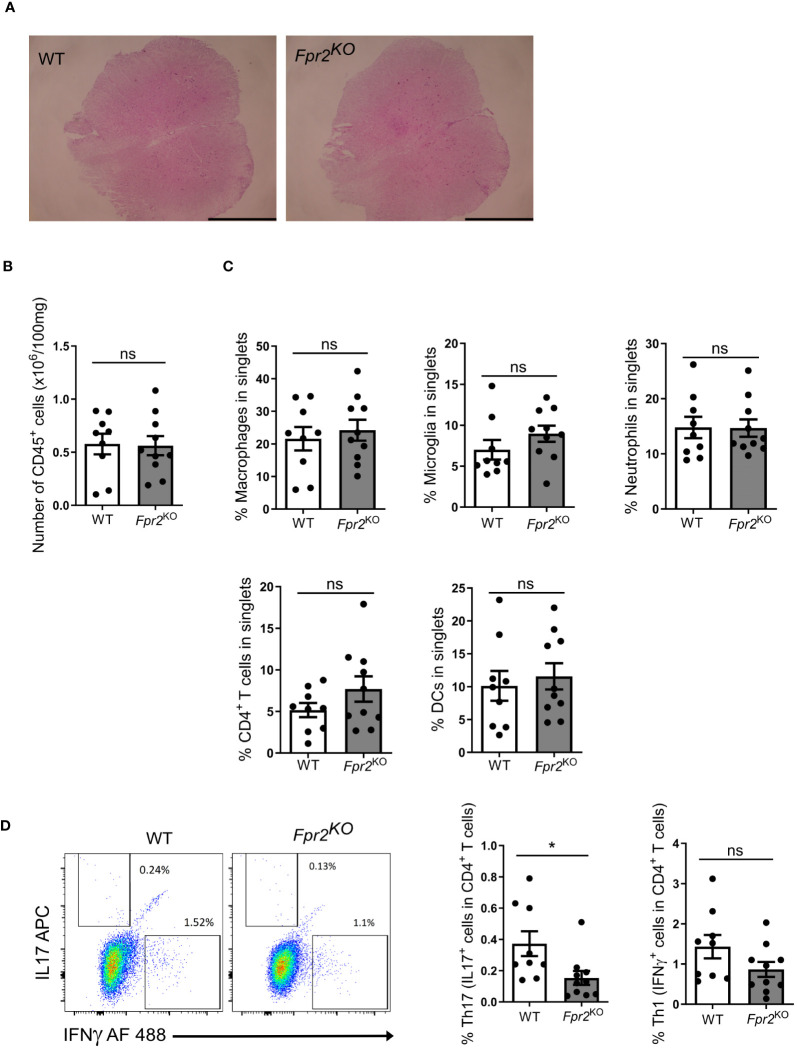
FPR2 deficiency was associated with decreased frequency of Th17 cells in spinal cords at the early stage of EAE. **(A)** Representative H&E staining images from spinal cords of WT and *Fpr2* ^KO^ mice subjected to EAE (day 12 post immunization). Scale bar: 1 mm. **(B-D)** Leukocytes were isolated from spinal cords of WT and *Fpr2* ^KO^ mice at day 12 after immunization and analyzed by flow cytometry. **(B)** The number of CD45^+^ leukocytes in spinal cords is shown. **(C)** The percentage in singlets of CD45^hi^CD11b^+^F4/80^+^ macrophages, CD45^int^CD11b^+^F4/80^+^ microglia, CD45^+^CD11b^+^Ly6G^+^ neutrophils, CD45^+^TCRβ^+^CD4^+^ T cells and CD45^+^CD11c^+^ DCs is shown. **(D)** Leukocytes from spinal cords were re-stimulated with MOG_35-55_ *in vitro*, and stained for intracellular IL17 and IFNγ, together with staining for CD4, and then analyzed by flow cytometry. The percentage of Th17 cells (IL17^+^ cells in CD4^+^ T cells) and Th1 (IFNγ^+^ cells in CD4^+^ T cells) is shown on the right; a representative flow cytometry plot is shown on the left. Data are mean ± SEM (n=9–10 mice per group). * P<0.05; ns, not significant.

Since the pathogenesis of EAE involves immune cell infiltration into the CNS (2), we characterized leukocyte populations in the spinal cords at the early effector phase of EAE (day 12 after immunization). No obvious differences in the inflammatory cell infiltrate were observed by H&E staining between the two genotypes (**Figure 2A**). Consistently, flow cytometry analysis revealed no significant difference in total number of CD45^+^ leukocytes (**Figure 2B**). Moreover, no difference was found in the frequency (percentage in singlets) of immune cell subpopulations, including CD4^+^ T cells (defined as CD45^+^TCRβ^+^CD4^+^), DCs (CD45^+^CD11c^+^), macrophages (CD45^hi^CD11b^+^F4/80^+^), microglia (CD45^int^CD11b^+^F4/80^+^), and neutrophils (CD45^+^CD11b^+^Ly6G^+^) (42), in the spinal cords of *Fpr2* ^KO^ mice and littermate WT controls (**Figure 2C**).

To further characterize the inflammatory response, we next conducted ex vivo antigen recall assays. To this end, leukocytes isolated from inflamed spinal cords were re-stimulated with MOG_35-55_, as described in the Methods, and the two cardinal cytokines involved in EAE-related inflammation, IL17A and IFNγ, were measured in culture supernatants. The release of both IL17A and IFNγ in recall assay cultures from *Fpr2* ^KO^ mice was reduced as compared to cultures from WT mice (**Supplementary Figure 2**). As IFNγ and IL17 can be produced by different leukocytes besides CD4^+^ T cells, we next determined the abundance of Th1 and Th17 cells, the major effector CD4^+^ T cells during EAE development (17, 43), in the antigen recall assay. To this end, we studied whether the presence of IFNγ- or IL17A-producing CD4^+^ T cells was altered due to FPR2-deficiency in the antigen recall assay. We found a decrease in the frequency of ex vivo re-stimulated Th17 cells (IL17^+^ cells in CD4^+^ T cells) deriving from the inflamed spinal cords of *Fpr2* ^KO^ mice, as compared to cells from WT controls (**Figure 2C**). In contrast, the frequency of ex vivo re-stimulated Th1 cells (IFNγ^+^ cells in CD4^+^ T cells) deriving from the spinal cord was not changed between WT and *Fpr2* ^KO^ mice (**Figure 2D**).

**Figure 3:**
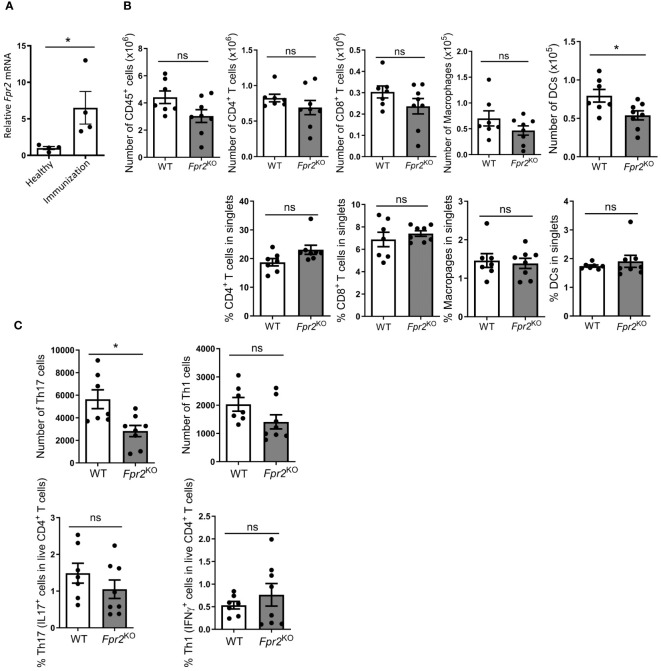
FPR2 deficiency resulted in decreased numbers of DCs and Th17 cells in draining lymph nodes at the pre-onset phase of EAE. **(A)** Relative *Fpr2* mRNA expression in the draining lymph node of healthy mice (not subjected to EAE) and mice subjected to EAE (day 7 post immunization), as assessed by qRT-PCR. The mRNA expression was normalized to *18S* and the gene expression of WT was set as 1 (n=4 mice per group). **(B, C)** Leukocytes were isolated from draining lymph nodes of WT and *Fpr2* ^KO^ mice on day 7 of the EAE model and analyzed by flow cytometry. The number of CD45^+^ leukocytes (B, top), as well as the numbers (B, top) or the percentage in singlets (B, bottom) of CD4^+^ (CD45^+^CD4^+^CD19^-^CD8^-^) T cells, CD8^+^ (CD45^+^CD8^+^CD19^-^CD4^-^) T cells, CD45^+^F4/80^+^ macrophages and CD45^+^CD11c^+^ DCs are shown (n=7–8 mice per group). **(C)** Isolated leukocytes were re-stimulated with MOG_35-55_ *in vitro*, and stained for intracellular IL17 and IFNγ, together with staining for CD4, and then analyzed by flow cytometry. Shown is the number of Th17 or Th1 cells (C, top), and the percentage of Th17 (IL17^+^ cells in live CD4^+^ T cells) or Th1 cells (IFNγ^+^ cells in live CD4^+^ T cells) (C, bottom) (n=7–8 mice per group). Data are mean ± SEM. * P<0.05; ns, not significant.

We subsequently studied the potential mechanism underlying the delayed EAE disease onset in *Fpr2* ^KO^ mice by focusing on the pre-onset phase of EAE development. Since the initiation of the T cell-specific immune responses occurs in the draining lymph nodes (dLN) (44, 45), and *Fpr2* mRNA expression in dLN is enhanced during EAE (**Figure 3A**), we examined whether FPR2 deficiency affected the initial immune priming. To this end, we performed flow cytometric analysis to examine different subsets of immune cells, including CD4^+^ T cells (CD45^+^CD4^+^CD19^-^CD8^-^), CD8^+^ T cells (CD45^+^CD8^+^CD19^-^CD4^-^), CD45^+^F4/80^+^ macrophages, as well as CD45^+^CD11c^+^ DCs, in the draining lymph nodes at day 7 post-immunization (pre-onset phase). *Fpr2* ^KO^ mice had significantly lower absolute numbers but not frequencies of DCs (defined as CD45^+^CD11c^+^ cells) in the dLNs during the pre-onset phase of the disease, whereas numbers and frequencies (as percentage in singlets) of other cell populations, e.g., CD4^+^ or CD8^+^ T cells and macrophages, were not significantly different between the two groups (**Figure 3B**). By performing an ex vivo antigen recall assay, we found a decrease in the number but not percentage (IL17^+^ cells in live CD4^+^ cells) of dLN Th17 cells from *Fpr2* ^KO^ mice, as compared to littermate WT mice (**Figure 3C**, left panels). In contrast, there was no difference between WT and *Fpr2* ^KO^ mice with regard to the numbers or percentage (IFNγ^+^ cells in live CD4^+^ cells) of dLN Th1 cells (**Figure 3C**, right panels). Together, FPR2 deficiency is associated with a reduction in DCs and Th17 cells in the dLNs during the pre-onset phase of EAE development, which may underlie the reduced abundance of Th17 cells in the inflamed spinal cords and thereby the delayed onset of EAE disease. These findings suggest that FPR2 contributes to Th17 cell differentiation in the course of EAE.

### 3.3 Impaired Th17 polarization due to FPR2 deficiency in dendritic cells

**Figure 4:**
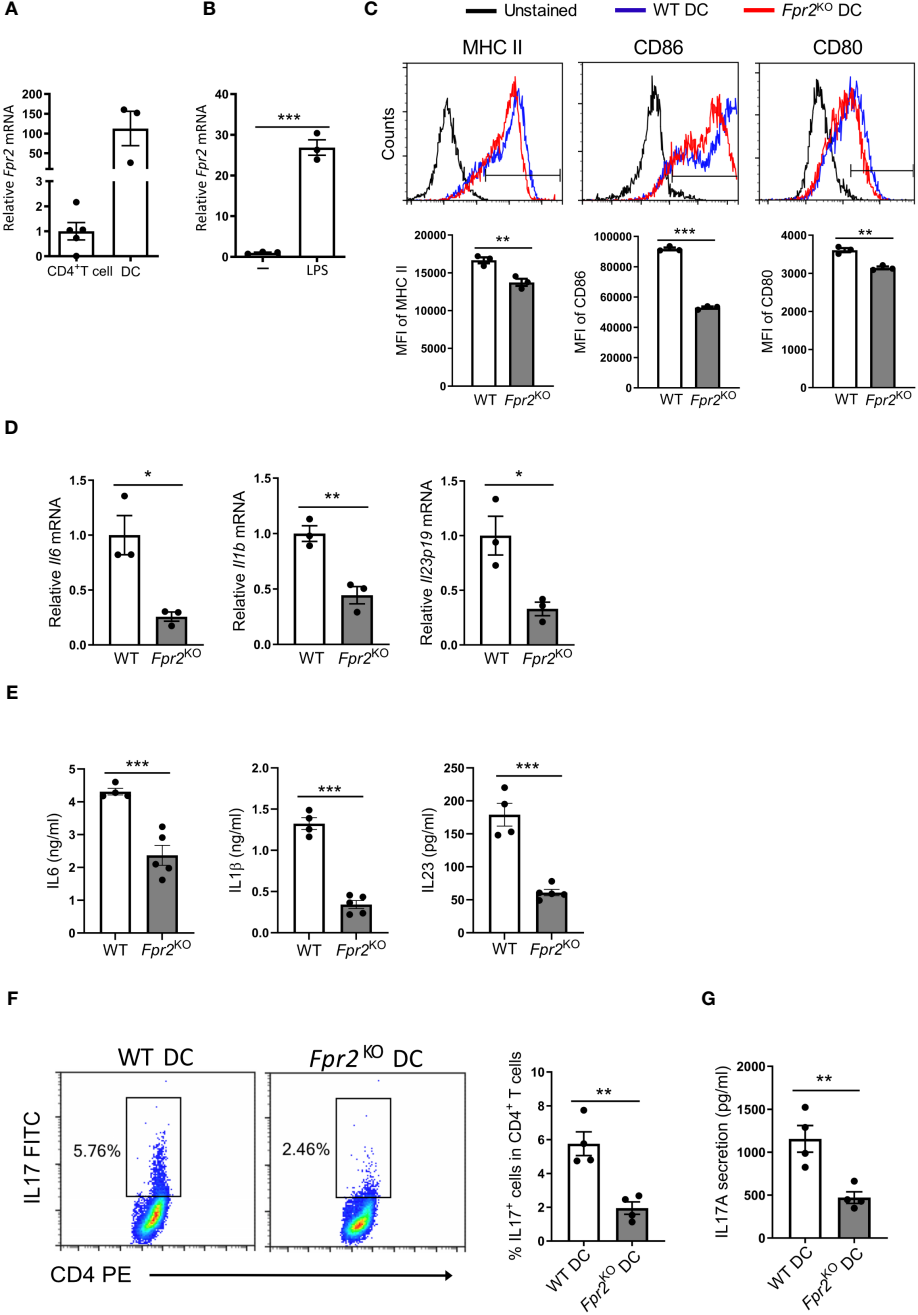
Decreased Th17 differentiation due to FPR2 deficiency in DCs. **(A)** Relative *Fpr2* mRNA expression in CD45^+^CD4^+^ T cells and CD45^+^CD11c^+^ DCs sorted from the draining lymph nodes of EAE mice on day 7 post-immunization. The mRNA expression was normalized to *18S* and the gene expression of CD45^+^CD4^+^ T cells was set as 1 (n=3–5 mice per group). **(B)** Relative *Fpr2* mRNA expression of BMDCs after overnight stimulation without **(-)** or with LPS, as assessed by qRT-PCR. The mRNA expression was normalized to *18S* and the gene expression of BMDCs in the absence of LPS was set as 1 (n=3 cultures). **(C)** Representative FACS histograms (top) and mean fluorescence intensity (MFI) (bottom) of MHC II (stained with anti-I-A^b^), CD86 and CD80 expression in WT or *Fpr2* ^KO^ CD11c^+^ BMDCs, activated with LPS for 3 hours, was assessed by flow cytometry (n=3 cultures). **(D)** Relative *Il6, Il1b, Il23p19* mRNA expression in WT or *Fpr2* ^KO^ BMDCs, activated with LPS for 3 hours, was assessed by qRT-PCR. The mRNA expression was normalized to *18S* and the gene expression in WT BMDCs was set as 1 (n=3 cultures). **(E)** IL6, IL1β, IL23 secretion in culture supernatants of WT or *Fpr2* ^KO^ BMDCs, activated with LPS overnight, was assessed by ELISA (n=4–5 cultures). **(F, G)** Naïve CD4^+^ T cells and WT or *Fpr2* ^KO^ BMDCs were co-cultured for 4 days in the presence of soluble anti-CD3, TGFβ1 and LPS to assess Th17 cell differentiation, as described in the Methods. The percentage of Th17 cells (IL17^+^ cells in CD4^+^ T cells) was studied by intracellular staining **(F)** and secreted IL17 production was detected in the culture supernatants by ELISA **(G)** (n=4 co-cultures). Data are mean ± SEM. Data in **(A, B, D, E)** are from one experiment; data in C,F,G are from one experiment representative of 2 experiments. *P<0.05, ** P<0.01, *** P<0.001.

Since FPR2 expression in DCs is much higher than in CD4^+^ T cells in the dLNs analyzed at day 7 after immunization (**Figure 4A**), we conducted mechanistic *in vitro* experiments to directly assess the contribution of FPR2 to the functional properties of bone marrow-derived dendritic cells (BMDCs). *Fpr2* mRNA expression was remarkably increased in mature BMDCs activated with lipopolysaccharide (LPS), compared to immature BMDCs (**Figure 4B**). We hypothesized that the lower Th17 differentiation potential observed in the dLNs of *Fpr2* ^KO^ mice is connected to the absence of FPR2 from DCs and hence a dysfunction of FPR2-deficient DCs. Indeed, flow cytometry analysis of LPS-activated BMDCs revealed reduced expression of both costimulatory molecules CD80 and CD86 as well as of MHC class II (MHC II) due to FPR2 deficiency (**Figure 4C**), in accordance to a previous report (46). Notably, moreover, mRNA expression of pro-inflammatory cytokines (*Il6*, *Il1b* and *Il23p19*) as well as expression of the respective proteins was reduced in FPR2-deficient mature DCs, as compared to WT mature DCs (**Figures 4D, E**). To investigate whether these changes in antigen presentation and cytokine production of DCs due to FPR2 deficiency directly affected Th17 differentiation, we employed an *in vitro* T cell differentiation system (47). Specifically, we co-cultured naïve CD4^+^ T cells with BMDCs in the presence of anti-CD3, human TGFβ1, and LPS for Th17 differentiation analysis (47). We compared Th17 cell development in the presence of FPR2*-*sufficient or -deficient BMDCs. Flow cytometry analysis revealed reduced Th17 differentiation in the presence of FPR2-deficient BMDCs, as compared to co-cultures with FPR2-sufficient BMDCs (**Figure 4F**). Consistently, we detected diminished IL17A secretion in the co-cultures with FPR2-deficient BMDCs, as determined by ELISA (**Figure 4G**). Taken together, these results demonstrate that FPR2-deficient BMDCs have impaired capacity to promote Th17 polarization.

### 3.4 Decreased Th17 differentiation due to FPR2 deficiency in DCs is mediated by altered NO production

**Figure 5:**
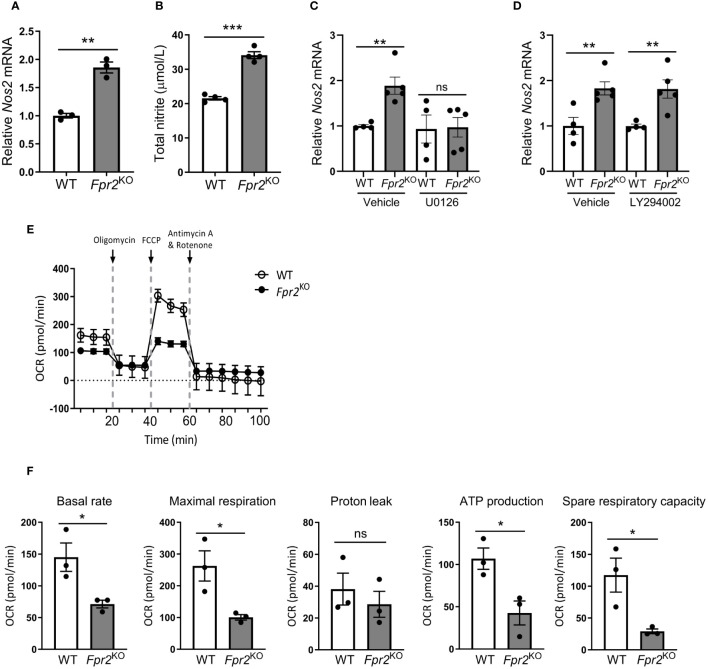
Deficiency of FPR2 increased DC nitric oxide production and reduced oxidative phosphorylation. **(A)** Relative expression of mRNA of *Nos2* (encoding iNOS) in WT or *Fpr2* ^KO^ BMDCs treated with LPS for 3 hours was determined by qRT-PCR (n=3 cultures). The mRNA expression was normalized to *18S* and the gene expression of WT BMDCs was set as 1. **(B)** Total nitrite level was determined in culture supernatants of WT or *Fpr2* ^KO^ BMDCs after stimulation with LPS overnight (n=4 cultures). **(C, D)** Relative *Nos2* mRNA expression in WT or *Fpr2* ^KO^ BMDCs, pre-treated with U0126 **(C)** or LY294002 **(D)** or vehicle (DMSO) for 30 mins followed by LPS treatment for 3 hours, was assessed by qRT-PCR. The mRNA expression was normalized to *Gapdh* and the gene expression of WT BMDCs pre-treated with vehicle was set as 1 (n=4–5 cultures). **(E, F)** Seahorse analysis was performed for studying oxygen consumption rate (OCR) following the manufacturer’s instructions, at baseline and after the administration of oligomycin (ATP synthase inhibitor), FCCP and Antimycin/Rotenone (Electron Transport Chain (ETC) inhibitor). In **(E)**, OCR of LPS-stimulated (6 hours) BMDCs from WT or *Fpr2* ^KO^ mice is shown, as measured by Seahorse analysis (n=3 cultures). In **(F)**, quantitative bar graphs of OCR for basal respiration rate, maximal respiration, proton leak, ATP production and spare respiratory capacity are shown (n=3 cultures). Data are mean ± SEM. Data in **(A, C, D)** are from one experiment; data in **(B)** are from one experiment representative of 3 experiments; data in **(E, F)** are from one experiment representative of 2 experiments.* P<0.05, ** P<0.01, ***p<0.001; ns, not significant.

Nitric oxide (NO) production modulates DC maturation (22) and suppresses Th17 cell polarization (26, 27). Interestingly, the mRNA levels of nitric oxide synthase 2 (*Nos2*), encoding the inducible NO synthase (iNOS), the primary NO-synthesizing enzyme in DCs, were significantly increased in FPR2-deficient mature DCs compared to WT counterparts (**Figure 5A**). This finding was further confirmed by measuring the total nitrite levels in culture supernatants, which suggested enhanced NO production by FPR2-deficient DCs (**Figure 5B**). Additionally, we investigated which signaling pathway is involved in the heightened *Nos2* expression resulting from FRP2 deficiency in DCs. Since the ERK (48, 49) and PI3K/Akt (50) pathways have been implicated in FPR2 signaling, we employed inhibitors of these pathways in LPS-treated DCs. Intriguingly, inhibiting the ERK but not the PI3K/Akt pathway abolished the increased *Nos2* expression due to FPR2-deficiency (**Figures 5C, D**). Therefore, the augmented *Nos2* expression due to FPR2-deficiency is mediated, at least in part, by the ERK pathway.

**Figure 6:**
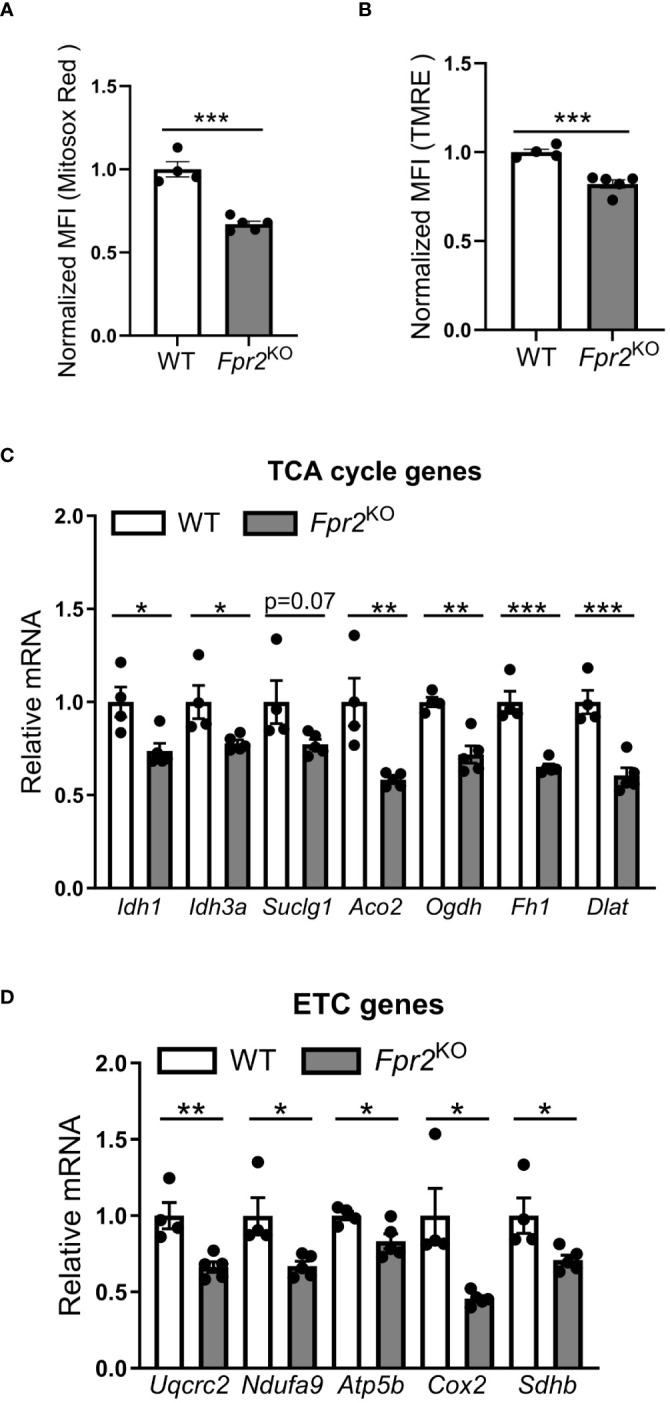
FPR2 deficiency diminished mitochondrial function. **(A, B)** Mean fluorescence intensity (MFI) of mitochondrial superoxide levels analyzed using MitoSox Red **(A)** or mitochondrial membrane potential analyzed using TMRE **(B)** in WT or *Fpr2* ^KO^ BMDCs, activated with LPS for 3 hours, as assessed by flow cytometry (n=4–5 cultures). The MFI was normalized and the MFI of WT BMDCs was set as 1. **(C, D)** Relative mRNA expression of TCA cycle genes (*Idh1, Idh3a, Suclg1, Aco2, Ogdh, Fh1, Dlat*), or ETC genes (*Uqcrc2, Ndufa9, Atp5b, Cox2, Sdhb*) in WT or *Fpr2* ^KO^ BMDCs, treated with LPS for 3 hours, was assessed by qRT-PCR (n=4–5 cultures). The mRNA expression was normalized to *Gapdh* and the gene expression of WT BMDCs was set as 1. * P<0.05, ** P<0.01, ***p<0.001.

Next, we explored alterations in cellular metabolism, as NO is known to modulate the metabolic switch during DC maturation (21). Therefore, we performed Seahorse XF extracellular Flux analysis to assess a role of FPR2 in oxygen consumption of DCs. Real-time metabolic analysis revealed a significant reduction in the oxygen consumption rate (OCR) in LPS-stimulated FPR2-deficient DCs, as compared to WT DCs (**Figures 5E, F**). Although DC maturation in response to LPS typically leads to a robust increase in glycolysis (51), we observed no difference in the extracellular acidification rate (ECAR) or mRNA levels of glycolytic genes including *Hk2, Ldha and Pkm2*, between FPR2-deficient and WT DCs (data not shown). Given the impaired OCR in DCs due to FPR2 deficiency, we further investigated mitochondria activity. Flow cytometry analysis revealed a significant decrease in MitoSox Red staining, indicative of reduced mitochondrial superoxide levels, in LPS-stimulated *Fpr2* ^KO^ DCs, compared to WT DCs (**Figure 6A**). Furthermore, mitochondrial membrane potential was impaired in LPS-stimulated FPR2-deficient DCs compared to WT counterparts as shown by TMRE fluorescence staining and flow cytometry analysis (**Figure 6B**). Additionally, the expression of genes related to the tricarboxylic acid (TCA) cycle and the electron transport chain (ETC) was diminished in LPS-stimulated FPR2-deficient DCs compared to WT DCs (**Figures 6C, D**). These results suggest that FPR2 plays a role in maintaining mitochondrial function and oxidative metabolism of DCs.

**Figure 7:**
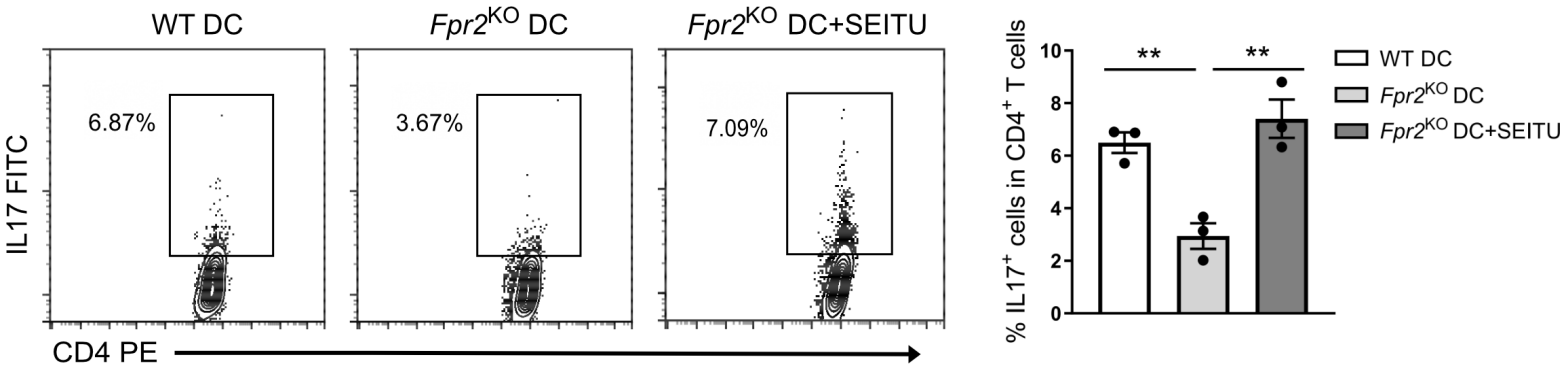
FPR2 in DCs promoted Th17 differentiation through inhibition of NO. Naïve CD4^+^ T cells and WT or *Fpr2* ^KO^ BMDCs were co-cultured for 4 days in the presence of soluble anti-CD3, TGFβ1 and LPS as well as in the presence or absence of the iNOS inhibitor SEITU. The percentage of Th17 cells (IL17^+^ cells in CD4^+^ T cells) was determined by intracellular staining. Data are mean ± SEM (n=3 co-cultures). Data are from one experiment representative of 2 experiments. ** P<0.01.

Next, to determine whether the diminished Th17 differentiation observed in the presence of *Fpr2* ^KO^ DCs is linked to the higher NO production, we inhibited iNOS using the potent inhibitor SEITU. We found that inhibition of NO production effectively reversed the inhibitory effect of FPR2 deficiency in DCs on Th17 cell differentiation, which was thereby completely restored (**Figure 7**). These findings suggest a key immunometabolic role of FPR2 signaling in DCs which, through decreased NO production, leads to enhanced Th17 differentiation.

## 4 Discussion

The Th17 subset of CD4^+^ T cells within the inflamed CNS has been widely implicated in both EAE and MS pathogenesis (52, 53). During the immune priming phase of EAE, Th17 differentiation is regulated by cytokines, primarily released by antigen-presenting cells, such as DCs (10, 17). Consistently, modifying DC function and their cytokine production influences EAE disease (54, 55). In the present study, we demonstrated that FPR2 signaling in DCs, through regulation of DC metabolism and inhibition of DC-derived NO production, promotes Th17 differentiation, thereby influencing the disease onset of EAE. Hence, FPR2 may act as a modifier of the early phase of inflammation in EAE.

The expression of FPR2 increases in dLNs following immunization, and the absence of FPR2 leads to a delayed onset of EAE. However, the disease attenuation at the onset of EAE caused by FPR2 deficiency is restored at later stages, suggesting that FPR2 primarily acts during the immune priming phase of EAE. Therefore, FPR2 on DCs may predominantly act in the lymph nodes during the initiation of the immune response in EAE. Accordingly, the reduced presence of Th17 cells in dLNs of *Fpr2* ^KO^ mice likely explains their lower frequency in inflamed spinal cords. However, a limitation of our study is that we used whole-body *Fpr2* ^KO^ mice, hence not allowing us to study exclusively the role of FPR2 on DCs *in vivo*. Previous studies have demonstrated that the adoptive transfer of MOG-loaded DCs before the onset of EAE leads to T regulatory cell-mediated protection against disease development (56, 57). Although our data suggest that FPR2 regulates DC function in the early phase of EAE, future investigations utilizing mice with DC-specific deletion of FPR2 would be required to further support this notion.

Recent research has shown that FPR2 is upregulated in the CNS during neuroinflammatory/neurodegenerative diseases like Alzheimer’s (58, 59) and CNS injury (60). However, while our study suggests a pro-inflammatory role for FPR2 at the initiation of EAE, FPR2 activation may also lead to anti-inflammatory and/or neuroprotective effects (60). In the EAE model, we conclude that the impact of FPR2 on disease onset is primarily associated with the function of FPR2 in dLNs, rather than in the inflamed CNS. These findings therefore highlight the diverse and context-dependent (e.g., cell- and tissue-dependent) role of FPR2 signaling on the inflammatory response.

Activation of DCs leads to production of inflammatory cytokines, and the initiation of adaptive immune responses. Deficiency of FPR2 in DCs was previously shown to lead to inadequate expression of maturation markers like CD86 and MHC II, along with reduced IL12 production (46), consistent with our study’s findings. In addition to these earlier findings, we have uncovered that absence of FPR2 signaling in DCs decreases the expression of cytokines, such as IL6, IL1β and IL23, that are critical for the differentiation of Th17 cells during the immune priming phase of EAE. This could be attributed to the observed reduced NO production in FPR2-expressing DCs upon LPS stimulation, compared to FPR2-deficient DCs, given that NO is required to restrain DC maturation and cytokine production (22). Consistent with this notion is our observation that inhibition of NO production effectively reversed the inhibitory effect of FPR2 deficiency in DCs on Th17 cell differentiation. While DC-derived NO appears to block Th17 differentiation, as shown in our study, NO produced by other cell types has been shown to support the development of Th17 responses in different inflammatory settings (61). This context-dependent role of NO highlights the complexity of its impact on immune regulation. Together, our study shows that FPR2 signaling in DCs promotes a cytokine environment that is conducive for Th17 cell development.

Activation of DCs by Toll-like receptor (TLR) agonists is associated with a progressive loss of mitochondrial activity (21). We found that FPR2-deficient DCs displayed decreased oxidative phosphorylation (OXPHOS) upon LPS stimulation. Although the inhibition of OXPHOS may result in higher reliance on glycolysis (21), we did not observe any differences in glycolysis between FPR2-deficient and WT DCs (not shown). Mitochondrial metabolic reprogramming in DCs depends on the specific DC subset and the context of stimulation (18). For instance, human plasmacytoid DCs (pDCs) have higher expression of the mitochondrial biogenesis regulator peroxisome proliferator-activated receptor gamma coactivator 1-alpha (PGC1-α) and the mitochondrial fusion promoter mitofusin 2, which support enhanced mitochondrial metabolism upon stimulation with TLR7/8 agonists. Conversely, human myeloid CD1c^+^ DCs decrease OXPHOS upon similar stimulation (62). Furthermore, the production of type I interferon (IFN-I) by human pDCs, following TLR9 activation, relies on glycolysis, while IFN-I production triggered by RIG-I-like receptor activation depends on OXPHOS (63). Based on our present findings, FPR2 appears to regulate mitochondrial metabolism in mouse BMDCs.

## 5 Conclusion

In conclusion, our study demonstrates that FPR2 signaling in DCs modulates DC metabolism and downregulates NO production, thereby promoting Th17 cell differentiation in the context of neuroinflammation.

## Data availability statement

The raw data supporting the conclusions of this article will be made available by the authors, without undue reservation.

## Ethics statement

The animal study was approved by the Landesdirektion Sachen, Germany and the Institutional Animal Care and Use Committee of the University of Pennsylvania, USA. The study was conducted in accordance with the local legislation and institutional requirements.

## Author contributions

J-HL: Conceptualization, Formal analysis, Investigation, Methodology, Supervision, Validation, Visualization, Writing – original draft. AN: Formal analysis, Investigation, Methodology, Validation, Visualization, Writing – original draft. K-JC: Writing – review & editing, Validation, Visualization. SG: Resources, Writing – review & editing, Investigation. OS: Writing – review & editing, Resources. GH: Project administration, Supervision, Writing – review & editing. TC: Conceptualization, Project administration, Supervision, Writing – review & editing.
