# Supplementary material for: Formyl peptide receptor 2 regulates dendritic cell metabolism and Th17 cell differentiation during neuroinflammation
Source: Front Immunol. 2024 Aug 1;15:1354074. doi: 10.3389/fimmu.2024.1354074 (PMC11324504; doi:10.3389/fimmu.2024.1354074)
Supplement: Supplementary file 1 [file DataSheet_1.pdf]

Supplementary Material

1.1 Supplementary Figures

Supplementary Figure 1

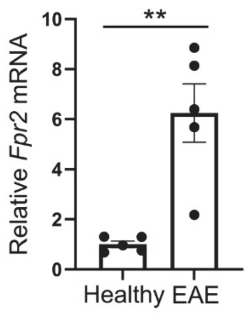

Supplementary Figure 1. Relative *Fpr2* mRNA expression in the spinal cords of healthy WT mice or WT mice subjected to EAE (at the early stage of disease; day 12 post-immunization), as assessed by qRT-PCR. The mRNA expression was normalized to *Gapdh* and the gene expression of healthy WT mice was set as 1 (n=5 mice per group). Data are mean ± SEM. **P<0.01.

Supplementary Figure 2

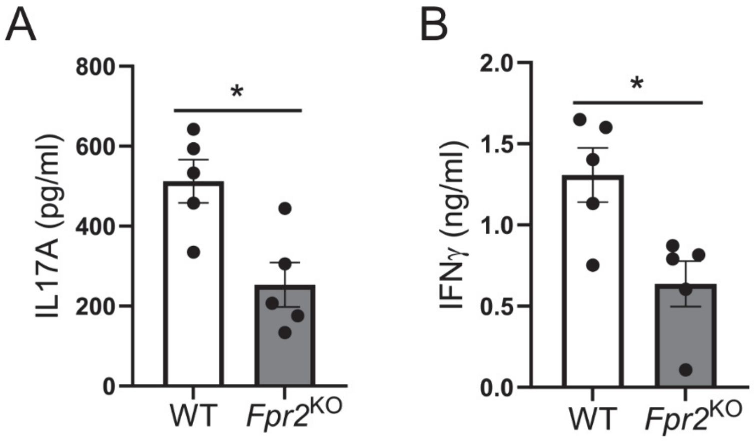

Supplementary Figure 2. Leukocytes isolated from spinal cords of WT and *Fpr2*^{KO} mice at day 12 after immunization were re-stimulated with MOG_{35-55} *in vitro* overnight, and culture supernatants were collected. Secreted IL17 (A) or IFNγ (B) in the culture supernatants was detected by ELISA. Data are mean ± SEM (n=5 mice per group). *P<0.05.
